# Insulin and Metformin Administration: Unravelling the Multifaceted Association with Mortality across Various Clinical Settings Considering Type 2 Diabetes Mellitus and COVID-19

**DOI:** 10.3390/biomedicines12030605

**Published:** 2024-03-07

**Authors:** Łukasz Lewandowski, Agnieszka Bronowicka-Szydełko, Maciej Rabczyński, Dorota Bednarska-Chabowska, Joanna Adamiec-Mroczek, Adrian Doroszko, Małgorzata Trocha, Krzysztof Kujawa, Agnieszka Matera-Witkiewicz, Edwin Kuźnik, Paweł Lubieniecki, Marcin Madziarski, Janusz Sokołowski, Ewa A. Jankowska, Katarzyna Madziarska

**Affiliations:** 1 Department of Biochemistry and Immunochemistry, Wroclaw Medical University, Chałubińskiego Street 10, 50-368 Wroclaw, Poland; lukasz.lewandowski@umw.edu.pl (Ł.L.); agnieszka.bronowicka-szydelko@umw.edu.pl (A.B.-S.); 2 Clinical Department of Diabetology and Internal Disease, Wroclaw Medical University, Borowska Street 213, 50-556 Wroclaw, Poland; dorota.bednarska-chabowska@umw.edu.pl (D.B.-C.); malgorzata.trocha@umw.edu.pl (M.T.); edwin.kuznik@usk.wroc.pl (E.K.); pawel.lubieniecki@usk.wroc.pl (P.L.); katarzyna.madziarska@umw.edu.pl (K.M.); 3 Clinical Department of Ophthalmology, Wroclaw Medical University, Borowska Street 213, 50-556 Wroclaw, Poland; joanna.adamiec-mroczek@umw.edu.pl; 4 Department of Cardiology, Faculty of Medicine, 4th Military Hospital, Wroclaw University of Science and Technology, Weigla 5 Street, 50-981 Wroclaw, Poland; adrian.doroszko@pwr.edu.pl; 5 Statistical Analysis Centre, Wroclaw Medical University, K. Marcinkowski Street 2–6, 50-368 Wroclaw, Poland; krzysztof.kujawa@umw.edu.pl; 6 Screening of Biological Activity Assays and Collection of Biological Material Laboratory, Wroclaw Medical University Biobank, Faculty of Pharmacy, Wroclaw Medical University, Borowska Street 221A, 50-556 Wroclaw, Poland; agnieszka.matera-witkiewicz@umw.edu.pl; 7 Clinical Department of Rheumatology and Internal Medicine, University Hospital, Borowska Street 213, 50-556 Wroclaw, Poland; marcin.madziarski@usk.wroc.pl; 8 Department of Emergency Medicine, Wroclaw Medical University, Borowska Street 213, 50-556 Wroclaw, Poland; janusz.sokolowski@umw.edu.pl; 9 Institute of Heart Diseases, Wroclaw Medical University, Borowska Street 213, 50-556 Wroclaw, Poland; ewa.jankowska@umw.edu.pl

**Keywords:** COVID-19, diabetes, mortality

## Abstract

Due to the molecular mechanisms of action of antidiabetic drugs, they are considered to be effective in the treatment of both COVID-19 and the post-COVID-19 syndromes. The aim of this study was to determine the effect of administering insulin and metformin on the mortality of patients with type 2 diabetes (T2DM) with symptomatic COVID-19 with the use of logistic regression models. The association between death and insulin and metformin was weak and could not be included in the multivariate model. However, the interaction of both drugs with other factors, including remdesivir and low-molecular-weight heparin (metformin), age and hsCRP (insulin), modulated the odds of death. These interactions hint at multifaceted (anti-/pro-) associations of both insulin and metformin with the odds of death, depending on the patient’s characteristics. In the multivariate model, RDW-SD, adjusted with low-molecular-weight heparin treatment, age, sex and K^+^, was associated with mortality among patients with COVID-19 and T2DM. With a 15% increase in RDW-SD, the risk of death increased by 87.7%. This preliminary study provides the foundations for developing further, more personalized models to assess the risk of death in T2DM patients, as well as for identifying patients at an increased risk of death due to COVID-19.

## 1. Introduction

According to the WHO, the prevalence of *SARS-CoV-2* reached approximately 662 million cases on the day of 16 January 2023, leading to 6 million deaths due to the development of the COVID-19 syndrome [1]. Although the pandemic has already passed, some people, especially those who suffered from full-blown COVID-19, may experience acute consequences of COVID-19, the so-called long COVID or post-COVID-19 syndrome (PCS), recognized by the WHO as the next epidemic of the 21st century [2]. This term refers to symptoms persisting for more than 3 months after COVID-19, causing long-term changes in single organs or multi-organ changes [1]. It is predicted that PCS may affect millions of people worldwide [1], mainly people with comorbidities [3], e.g., type 2 diabetes mellitus (2TDM). The symptoms of PCS are difficulty with concentration, cognitive dysfunction, amnesia, depression, fatigue and anxiety [3], and the risk factors for the persistence of neuropsychiatric symptoms in PCS are older age, female sex and the severity of comorbidities, e.g., diabetes [4], which is often associated with tachycardia, sarcopenia, microcirculatory dysfunction or organ damage [5]. PCS is therefore a phenomenon that affects life expectancy.

The study reported in this manuscript focuses on diabetes, which was shown to develop de novo in patients suffering from COVID-19 [4]. Diabetes is estimated to be associated with approximately 15% of patients suffering from severe COVID-19. The mortality rate in COVID-19-positive diabetic patients was reported to be 2- to 3-fold higher compared to COVID-19-negative diabetic patients [5]. The literature (studies and meta-analyses) shows higher mortality in the COVID-19-positive diabetic population compared to the COVID-19-positive non-diabetic population [6,7,8,9]. Moreover, optimal diabetic control (in the case of T1DM/T2DM) was associated with better outcomes and fewer comorbidities among COVID-19-positive patients [10]. Such statistics account for the urge to partake in therapeutic intervention [5]. However, the information on the COVID-19-associated diabetic cases and their responses to drug treatment are scarce. Research into new strategies in its treatment and prevention may improve the quality of medical decisions in terms of mortality, exacerbation and optimal response to drugs [11], and shed some light on the metabolic alterations in these patients.

A handful of clinical studies aimed to combine the classic anti-viral and anti-inflammatory drugs in one treatment scheme in order to combat COVID-19 [11]. However, since the molecular action of agents used in antidiabetic treatment is multidimensional and may possibly modulate the course of COVID-19 and its related oxidative stress and cytokine storm [12,13,14,15], interactions with antidiabetic treatments ought to be taken into account when analyzing multidimensional models used in more complex studies. To our knowledge, no studies analyzed the difference in the odds of mortality associated with antidiabetic treatment in the context of the simultaneous effects of the coexisting covariates (comorbidities, patient characteristics and demography, and biochemical parameters). The lack of such an investigation renders the one-dimensional studies prone to generate false assumptions, owing to the aforementioned multifaceted action of antidiabetic drugs and the *SARS-CoV-2* affinity towards cell membrane proteins [5,13,15,16,17,18], often acting as intrinsic cell-to-cell messengers. 

The aim of this preliminary study was to explore the mortality-wise effect of insulin and metformin administration in the European (Polish) model of the COVID-19-positive diabetic population sample composed of all patients admitted to the Temporary COVID-19 Hospital in Wroclaw, Poland. Typically, patients transferred to this specific hospital were characterized by an increased risk of in-hospital mortality due to increased COVID-19 severity. Along with the estimated effect of insulin and metformin on survival, their interactions with other factors (including other agents) were studied (in the form of second- or third-degree interactions) to check for synergies in modulating the mortality rate. Only significant interactions were reported and further analyzed, as opposed to the less optimal process of adjusting the findings with a pre-assumed set of patient features. This study design was chosen so as to give a foundation for future, more patient- and treatment-oriented risk assessment models that would be validated on bigger, stratified, diabetic population samples. These tools would enable the identification of patients with a higher risk of death from COVID-19. This targets those for whom analysis of the values of the selected laboratory and demographic parameters, and taking into account the treatment methods for both T2DM and COVID-19, would prove to be at-risk in the context of assessment of the outcome of the disease.

## 2. Materials and Methods

### 2.1. Inclusion/Exclusion Criteria and the Population from Which the Sample Was Taken

A retrospective analysis was performed on 430 medical records of patients with type 2 diabetes and *SARS-CoV-2* infection (the total number included data from 2151 patients) hospitalized between February 2020 and June 2021 at the University and Temporary COVID-19 Hospital organized by the University Medical Hospital in Wroclaw (Poland), which were collected as part of the COLOS registry (COronavirus in LOwer Silesia). All patients admitted at the hospital due to COVID-19 symptoms tested positive for the presence of *SARS-CoV-2* in nasopharyngeal swab specimens using the RT-PCR method (via reverse transcription and polymerase chain reaction), which was recommended by the World Health Organization (WHO) [19]. The tests were performed using the two-gene test (ORF1ab, N) SARS-CoV-2 Real Time PCR LAB-KIT (BIOMAXIMA, Lublin, Poland), using the QuantStudio 6 Flex device (Applied Biosystems, Warsaw, Poland). Isolation of nukelic acids was performed on a Magna Pure 96 apparatus (Roche, Basel, Switzerland). The observation period lasted from the day of hospital admission to the day of discharge or death. The study protocol was approved by the Bioethical Committee and Ethics Committee of the Medical University in Wroclaw, Poland (No: KB-444/2021), and permission was granted for the publication of anonymized data. All patients provided written consent for admission into the study, which stipulated that the results may be used for research purposes. This study was in accordance with Helsinki declaration.

**Figure 1:**
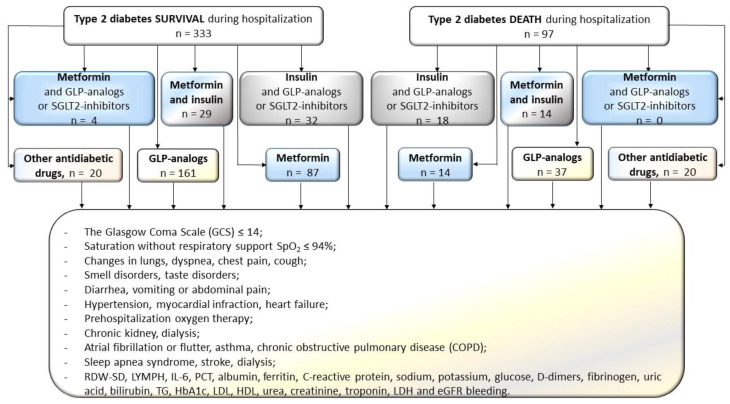
Diagram illustrating the frequency of drug use in regard to antidiabetic treatment in the diabetic patients with COVID-19. A set of features that were analyzed among the two survival statuses is given as a reference.

The dataset included demographic information (sex, age), concurrent conditions, performed procedures, vital signs and laboratory test results during hospitalization. This study accounted for the following variables determined before hospital admission: gender; type 2 diabetes therapies used before hospital admission, including metformin, insulin, metformin and insulin, insulin and another oral antidiabetic drug, GLP agonists (semiglutide or dulaglutide injected subcutaneously) or metformin and another oral antidiabetic drug (SGLT-2 inhibitors); the Glasgow Coma Scale (GCS) ≤ 14; oxygen saturation without respiratory support SpO_2_ ≤ 94%; lung lesions (typical of *SARS-CoV-2* infection, observed under the CT scan); dyspnea; chest pain; cough; smell disorders; taste disorders; diarrhea, vomiting or abdominal pain; hypertension; hemorrhage (gastrointestinal, respiratory, intracranial, genital, urinary); myocardial infraction; heart failure; prehospitalization oxygen therapy; chronic kidney; atrial fibrillation or flutter; asthma; chronic obstructive pulmonary disease (COPD); sleep apnea syndrome; stroke; dialysis; red cell distribution width—standard deviation (RDW-SD); concentration of lymphocyte (LYMPH), interleukin 6 (IL-6), procalcitonin (PCT), albumin, ferritin, C-reactive protein, sodium, potassium, glucose, D-dimers, fibrinogen, uric acid, bilirubin, diacylglycerides (TG), glycated hemoglobin (HbA1c), low-density lipoprotein (LDL), high-density lipoprotein (HDL), urea, creatinine, troponin and lactate dehydrogenase (LDH); and estimated glomerular filtration rate (eGFR) calculated based on the Modification of Diet in Renal Disease (MDRD) Study Equation [20]. The analysis also took into account features that were observed upon clinical assessment or introduced (in the case of iatrogenic factors) during the hospitalization. Apart from antidiabetic agents for which the administration was continued during the hospitalization, therapeutic agents were introduced in the treatment upon admission of the study participants to the Temporary COVID-19 Hospital. The initial dataset included variables such as stroke, revascularization (PCI or CABG), features of pulmonary obstruction or pneumonia, shock (hypovolemic, cardiogenic, septic), hemorrhage (gastrointestinal, respiratory, intracranial, genital, urinary), myocardial infarction, venous thromboembolism (embolism, deep vein thrombosis, embolism and thrombosis), new neurological disorders, smell disorders and taste disorders, convalescent plasma, remdesivir and acetylsalicylic acid. The study group was divided based on survival during the hospitalization. The study participants were not further monitored in terms of mortality after their hospitalization had ended. In each of these two groups, the method of treating type 2 diabetes (T2DM) was identified, according to Figure 1.

### 2.2. Statistical Methods

Data preprocessing and visualization were performed with Python 3.10.7 (packages: pandas 1.4.4, numpy 1.21.4, matplotlib 3.5.3, seaborn 0.11.2). Statistics were employed with use of Statistica 13.3 on license by Wroclaw Medical University. Characteristics of the population sample were performed with use of the Mann–Whitney U and χ^2^ tests. In case of low (<5) estimated count in any contingency table cell, Yates correction for continuity was applied. The normality assumption was checked with use of the Q–Q plots and the Shapiro–Wilk test.

Odds of death were analyzed with use of logistic regression models. Expert analysis of the dataset by a multidisciplinary team (consisting of medical doctors, biochemists, laboratory medicine professionals, statisticians and a data scientist) led to drawing candidates to be featured in the initial set used for deriving the optimal multivariate model. A subsequent analysis of the % of missing data led to obtaining the initial set of effects (variables) used for modeling. Some continuous effects were log-transformed so as to meet the assumption of linearity vs. log(Odds) tested with the Box-Tidwell test. To make the intercepts from the model resemble real-life conditions, continuous variables were centered at selected, typical values of the entire population sample from which the data used in this study was extracted. At the beginning of the analysis, univariate odds ratios (ORs) were analyzed (Table A1). Subsequently, the optimal model was derived from the initial set of the variables with use of the stepwise elimination (*p* cut-off for inclusion/exclusion: 0.05) iterative process (Table A2). Interaction analysis was the final part of the study, in which significant interactions (Table A3) and the effects that took part in them were incorporated into the models, based on the type 1 likelihood ratio (LR) test aimed to assess whether any interaction would be more explanatory in the context of the odds of death, compared to the naïve model which was not based on any variables. The interactions that were significant were incorporated in models reported in this manuscript. The mentioned models contained the intercepts, interaction terms and the features (effects) taking place in these interactions—as per the good practice in data modeling.

## 3. Results

### 3.1. Characteristics of the Population Sample Used in this Study

Among patients with T2DM hospitalized due to symptomatic COVID-19, significant differences affecting survival were observed among the following criteria described upon hospital admission: sex (women survival: 48.35%, women death: 34.58%), SpO_2_ ≤ 94% (survival: 51.04%, death: 67.39%), hemorrhage (survival: 5.41%, death: 14.95%), myocardial infarction (survival: 14.11%, death: 28.97%), heart failure (survival: 21.62%, death: 28.97%), prehospitalization oxygen therapy (survival: 47.75%, death: 64.49%), chronic kidney diseases, CKDs (survival: 18.02%, death: 29.91%). Furthermore, the type of therapy used in type 2 diabetes also influenced the differences in patient survival: using only metformin (survival: 26.13%, death: 13.08%) versus insulin and other antidiabetic drugs (survival: 9.61%, death: 16.82%) had different results, which, of course, stemmed from the stage of advancement of T2DM. Table 1 shows the baseline demographic and clinical characteristics of the study participants. Non-survivors differed from the survivors in terms of the following laboratory-measured parameters: RDW-SD, LYMPH, IL-6, PCT, albumin, ferritin, hsCRP, potassium, glucose, eGFR, urea, creatinine, LDH and troponin, as shown in Table 1.

Among patients who died during hospitalization, there were more frequent occurrences of hypovolemic shock, cardiogenic shock or sepsis (survival: 3.90%, death: 42.06%). Hemorrhagic episodes were more common (survival: 5.41%, death: 14.95%). Moreover, non-survivors showed/underwent the following features more frequently: pulmonary obstruction or pneumonia (survival: 55.86%, death: 79.44%), myocardial infarction (survival: 1.80%, death: 5.61%) and revascularization procedures such as percutaneous coronary intervention (PCI) or coronary artery bypass grafting (CABG) (survival: 1.80%, death: 5.61%). As per treatment-wise choices, non-survivors were characterized with higher intakes of acetylsalicylic acid, most presumably as a result of the more severe course of COVID-19 (survival: 26.73%, death: 38.32%), as shown in Table 1. There were no statistically significant differences in the administration of convalescent plasma or remdesivir between the two groups.

### 3.2. Univariate Modulation of the Odds of Death

**Figure 2:**
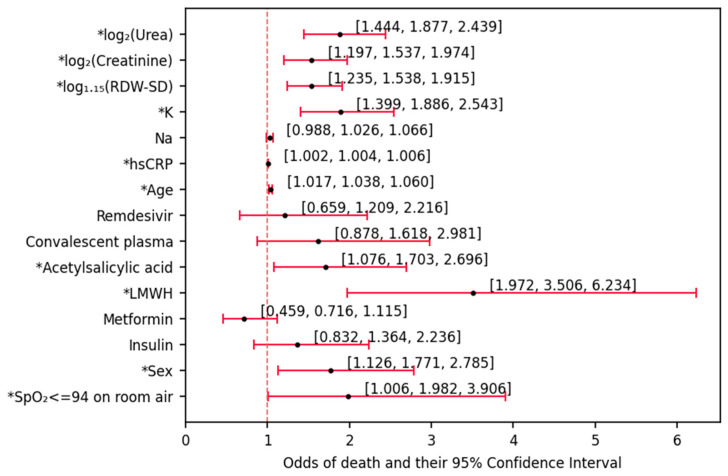
Univariate odds ratios (ORs) of death, associated with the analyzed set of effects (variables). These ORs show the fold change in odds of death associated with each effect, individually (e.g., the baseline odds for each effect would refer solely to this effect), * significant results (*p* < 0.05).

According to the univariate analysis (Figure 2), 10 out of 15 variables proved to have significant influence on the odds of death. Having the SpO_2_ lower than 94 increased the odds of death by 98.2% (*p* ≈ 0.048). Male individuals were 77.1% more likely to die compared to female (*p* ≈ 0.013). Each one-year increase in age would increase these odds by 3.8% (*p* < 0.001). Among the used drugs analyzed in this study, two out of six proved to be associated with higher odds of death: LMWH (by 3.506-fold, *p* < 0.001) and acetylsalicylic acid (by 70.3%, *p* ≈ 0.023). The administration with other agents (remdesivir, convalescent plasma, metformin, insulin) was found to be, per se, insignificant in terms of modulation of these odds. Each subsequent increase in hsCRP (by 1 mg/L) and potassium (by 1 mmol/L) increased the odds by 0.4% (*p* ≈ 0.001) and 88.6% (*p* < 0.001), respectively. Each 15% increase in RDW-SD increased the odds by 53.8% (*p* < 0.001), while each 2-fold increase in creatinine and urea increased these odds by 53.7% (*p* ≈ 0.001) and 87.7% (*p* < 0.001), respectively.

Although the calculated odds ratios (ORs) for administration with metformin and insulin were 0.716 and 1.364, respectively, these insights were insignificant in relation to the entire population (*p* > 0.05, as the confidence interval exceeded the OR = 1 line shown in Figure 2).

### 3.3. Multi-Effect Modulation of the Odds of Death According to the Model Derived with the Stepwise Elimination Model

The derived multi-effect model (Table 2) consisted of the following effects: potassium (*p* < 0.001), sex (*p* ≈ 0.014), RDW-SD (*p* ≈ 0.002), age (*p* ≈ 0.002) and LMWH treatment (*p* ≈ 0.002).

**Figure 3:**
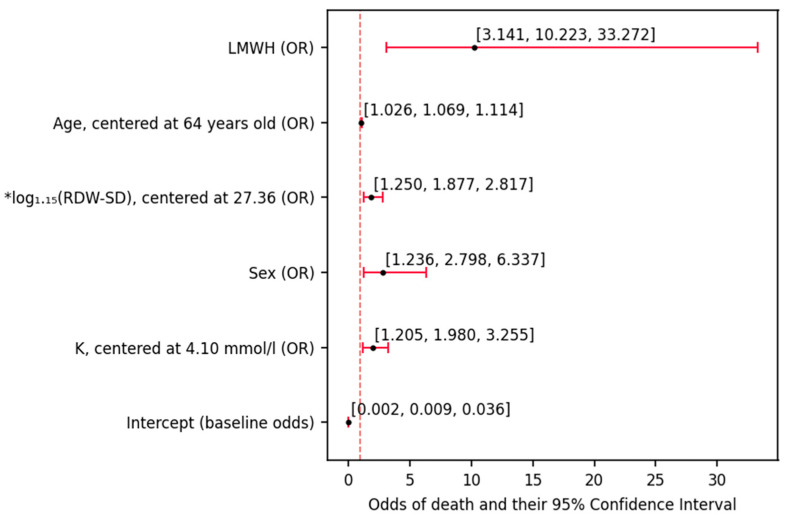
Multi-effect odds ratios (ORs) of death associated with the model derived through iteration (stepwise elimination). The baseline odds represent the odds of death among individuals with the following characteristics: female, neither low-molecular-weight heparin (LMWH), insulin nor remdesivir treatment, 45.78 RDW-SD, 4.10 mmol/L K, aged 64 years old. The ORs show the fold change in baseline odds ratios (ORs) associated with each effect (variable). Significant findings (*p* < 0.05) were marked with ‘*’.

The baseline odds (estimated for a 64-year-old female with RDW-SD 45.78 and K 4.10 mmol/L, under no LMWH treatment) were 0.009, suggesting very high pro-survival tendency (9 deaths per 1000 individuals). These odds would be modulated (Figure 3) by the following effects: K (by 98% per each 1 mmol/L increase), male sex (by 2.798-fold), age (by 6.9% per each 1-year increase), RDW-SD (by 87.7% per every 15% increase) and administration of LMWH (by 10.223-fold).

The baseline odds of death were established for designated representatives. This allows for the simultaneous application of all variables in the model to calculate the odds ratio for death concerning specific characteristics (LMW, age, RDW-SD, sex, potassium centered at 4.10 mmol/L) relative to the baseline person. For a 64-year-old woman not taking LMWH, with an RDW-SD value of 45.78 fL, every increase in potassium by 1 mmol/L from the baseline value of 4.10 mmol/L increased the odds of death by 98%. Additionally, for every one-year increase in age beyond 64 years without taking LMWH, RDW-SD at 45.78 fL, and potassium concentration at 4.10 mmol/L, there was a 6.9% increase in the odds of death.

The baseline odds (estimated for a 64-year-old female with RDW-SD 45.78 and K 4.10 mmol/L, under no LMWH treatment) was 0.009, suggesting very high pro-survival tendency (9 deaths per 1000 individuals). These odds would be modulated (Figure 3) by the following effects: K (by 98% per each 1 mmol/L increase), male sex (by 2.798-fold), age (by 6.9% per each 1-year increase), RDW-SD (by 87.7% per every 15% increase) and administration of LMWH (by 10.223-fold).

### 3.4. Significant Contrasts. Part 1: How Both LMWH and Remdesivir Modulated the Effect of Metformin Intake on the Odds of Death

**Figure 4:**
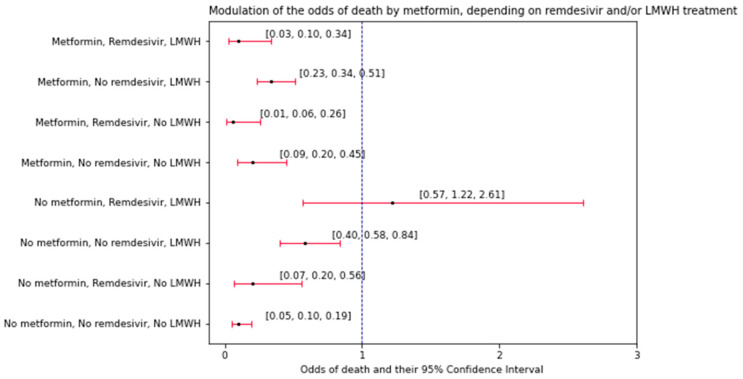
Estimated baseline odds of death depending on administration of metformin and/or remdesivir and/or low-molecular-weight heparin (LMWH).

According to the model, the modulation of the odds of death by metformin treatment were dependent on administration of both LMWH and remdesivir, although the baseline odds differed between different types of treatment (Figure 4). The stratum administered with LMWH and remdesivir but no metformin was the only one that showed a baseline ratio approximately equal to 1 (*p* ≈ 0.617), indicating nearly identical odds of death and survival. The other strata (Figure 4) showed significantly higher baseline odds of survival compared to the odds of death.

Based on the estimations from the model among the stratum that did not undergo either remdesivir or LMWH treatment, individuals who were administered with metformin would show 2.10-fold higher odds of death compared to individuals who were not administered with metformin. This fold difference would be approximately 3.54-fold lower (*p* ≈ 0.036) in the stratum administered with LMWH (in patients with or without remdesivir administration), or 6.93-fold lower (*p* ≈ 0.011) in the stratum administered with remdesivir (in patients with or without LMWH administration). Interestingly, LMWH and remdesivir did not significantly affect each other in the way that they modulated the effect of metformin intake on the odds of death (β = 0.069, 95% CI: 0.004–1.32, *p* ≈ 0.076). Based on the baseline odds of death for different patient characteristics, metformin intake was associated with higher odds of death under no treatment with LMWH and remdesivir, although it would promote survival if LMWH and/or remdesivir had been administered during hospitalization (Figure 4).

### 3.5. Significant Contrasts. Part 2: Insights into Aging. Inflammation and its Mutual Effect on How Insulin Affected the Odds of Death

Similar to the previous subsection, the interactions selected for further exploration were based on their significance upon applying the likelihood ratio (LR) test. To fully understand the interaction between age, hsCRP and insulin on the odds of death, one needs to assume that the baseline individual who would be referred to in this subsection was aged 64 with hsCRP equal to 48.88.

Interestingly, age- and inflammation-associated changes in the odds of death in the baseline individuals would depend on insulin administration (Table 3). Among patients not administered with insulin, every subsequent one-year increase in age or one-unit increase in hsCRP would cause, respectively, 0.6% or 6.1% increases in the odds of death (*p* < 0.001). Conversely, these age and inflammation-wise changes in odds of death were not observed among patients administered with insulin.

**Figure 5:**
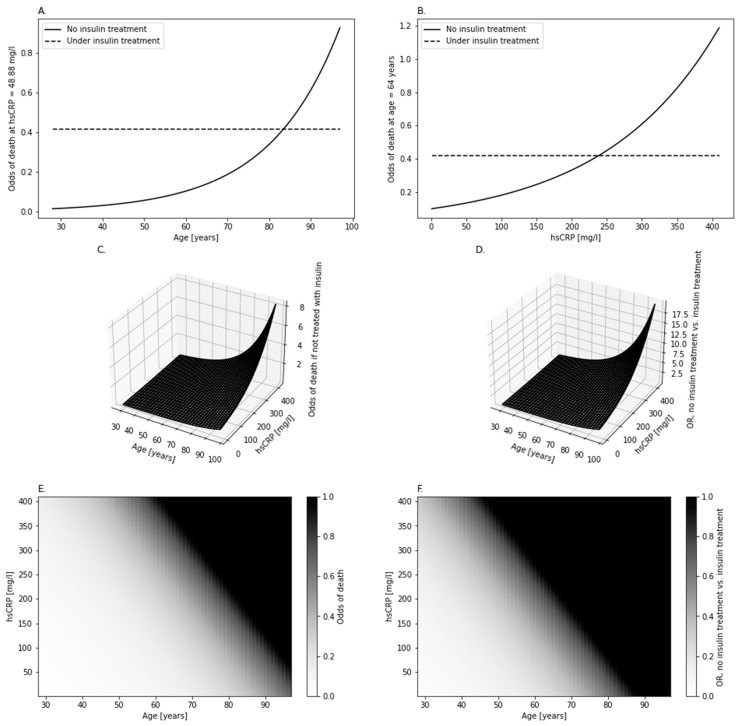
The three-way modulation of the odds of death by insulin, age and inflammation (high-sensitivity CRP—hsCRP). Plots (**A**,**B**) show how age (**A**) or hsCRP (**B**) change the odds of death depending on the administration of insulin. Plot (**C**) shows how age and hsCRP, together, change the odds of death among individuals not administered with insulin. Plot (**D**) presents the not administered/administered (with insulin) OR depending on both age and hsCRP. Plots (**E**,**F**) are heatmaps created from plots (**C**,**D**), showing when the probability of death is higher than the probability of survival (odds > 1 shown in black plot (**E**)) or when the individuals not administered with insulin are of the higher odds of death compared to individuals who were administered with insulin (OR > 1 shown in black, plot (**F**)).

Let us further assume that the baseline individuals were not administered with insulin. Based on the model (Table 3), this stratum would show approximately 3.16-fold higher odds of death if insulin were administered (*p* ≈ 0.001). This occurrence stemmed from different baseline odds of death depending on insulin administration (0.4174 if administered vs. 0.1319 if not). However, as both age and hsCRP modulated these odds only in individuals not administered with insulin (Figure 5A,B), there are age and hsCRP-related characteristics (Figure 5D,F) that would not only render the odds in this group higher than the 0.4174 (the baseline odds for patients administered with insulin), but also make death more probable than survival (odds > 1) among this sole group (e.g., not administered with insulin), as shown in Figure 5C,E.

### 3.6. Significant Contrasts. Part 3: The Association between Death and LMWH Treatment Differed Depending on Age

**Figure 6:**
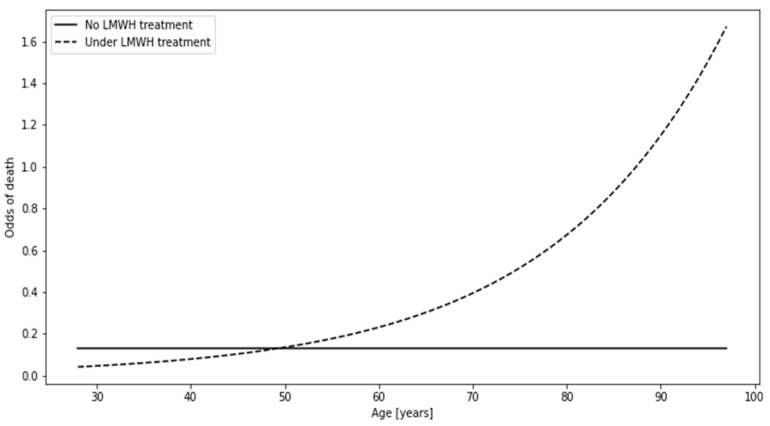
The relationship between the odds of death and the patient’s age depending on the administration of low-molecular-weight heparin (LMWH).

The baseline individual referred to in this subsection was assumed to be 64 years old (Table 4). According to the model, such a patient would most probably survive (odds < 1), posing odds of death approximately 0.131 under no LMWH treatment or approximately 0.286 if LMWH was administered (OR ≈ 0.456, *p* ≈ 0.022). As age, per se, would significantly alter the odds of death only among individuals administered with LMWH (5.5% increase of the odds with each one-year increase in age), the administered/not administered OR of death would be increasing by 6.1% with every one-year age increase above 64 years old (*p* ≈ 0.019), as shown in Figure 6.

## 4. Discussion

COVID-19, as many other inflammation-driving diseases/syndromes, induces the excessive production of inflammatory cytokines (‘cytokine storm’) leading to activation of CD4- and CD8-positive lymphocytes. This dysregulation leads to development of various aforementioned comorbidities, often leading to increased mortality [4,21]. In this study, non-survivors were characterized by male sex; higher age; the following traits upon their admission: SpO_2_ ≤ 94%, undergoing oxygen therapy (in many cases due to the severity of the disease) and hemorrhage; and the following traits associated with their medical history: heart failure, heart infarction and chronic kidney disease. These findings were in line with the literature, which links higher mortality in patients suffering from COVID-19 and T2DM with male sex [22] heart failure, chronic kidney disease [23], hyperglycemia (poor diabetic control) [24], lower oxygen saturation [25] or heart infarction in the past [26]. Another study lists cardiovascular diseases (such as coronary artery disease) and stroke as other death-promoting factors [27]. Moreover, one of the aforementioned studies [21] also showed that death during hospitalization was more frequent among the patients who had developed the following during the hospitalization: hypovolemic shock, cardiogenic shock, heart infarction, sepsis, chronic obstructive pulmonary disease (COPD) or other inflammatory pulmonary syndromes [22]. However, to our knowledge, the literature does not sufficiently cover the topic of risk modeling in these patients (COVID-19 and T2DM), as it focuses on analyzing the effects of different factors on mortality with use of the models. Albeit the models correct the estimated values (odds, risk etc.) for characteristics such as sex, age and comorbidity, they do not utilize nor enable exploring the interactions between these factors, let alone exploring the interactions between the drugs administered during the hospitalization. An example of such a study carried out on the COVID-19- and T2DM-positive stratum [28] showed that COPD increased the odds of death. However, this effect was not further explored. In this situation, one would be left to their own interpretation, not knowing whether the COPD-associated increase in mortality would be further modulated by any comorbidity, requiring the use of a different set of patient characteristics depending on these comorbidities. Explorative interaction analysis could prove an answer to this question, possibly revealing the population strata that does not show an association between COPD and higher mortality. This and similar musings led to the conceptualization of our preliminary study [29,30,31,32]. 

The aim of this study was dichotomic. Univariate analysis and multivariate AI-assisted extraction of the key factors associated with the odds of death in the COVID-19- and T2DM-positive patient stratum acted as a prelude to exploring whether the pro-fatal effect of antidiabetic and anti-viral drugs administered during hospitalization was affected by any patient-specific characteristic upon admission.

Nearly all of the available parameters were associated with the odds of death based on univariate (not adjusted by any other variables) logistic regression analysis. The analysis revealed that older age and male sex (unsurprisingly) were positive factors for estimating mortality odds. Having an oxygen saturation under the physiological values (95%–100%), likewise, was positively associated with a fatal outcome. LMWH and acetylsalicylic acid were iatrogenic factors promoting in-hospital death. Moreover, among the laboratory parameters, hsCRP, K, creatinine, urea and RDW-SD were positive indicators of higher odds of death. While it is logical that the inflammation- and diuresis- related parameters would be associated with higher severity of the disease [29,30,31], the anisocytosis parameter (RDW-SD) may not be a first-pick candidate for a poor outcome predictor. In clinical practice, RDW is a predictor of outcome in critically ill and septic patients (an increase in RDW is caused by an increase in the number of old red blood cells, which have a lower volume). Several meta-analyses have demonstrated the association between RDW and the risk of mortality in patients with COVID-19 [33] and proved the important role of RDW in predicting prognosis [34]. RDW-SD has also been shown to be a strong independent predictor of infection severity and death in COVID-19 patients: an RDW-SD ≤ 43 showed no risk of death, while RDW-SD > 47 indicated severe disease and a high risk of mortality [35]. If the RDW-SD value would fall in the range of 43 < RDW-SD ≤ 47, the course of the disease would be severe, but the risk of death was low. Therefore, it seems likely that determining the value of RDW may prove important in undertaking early intervention to reduce mortality in COVID-19 patients, especially in the case of limited resources. However, so far, the role of RDW has not been previously demonstrated in patients with COVID-19 and type 2 diabetes. Therefore, univariate logistic regression analysis showed that age, male sex and RDW-SD are positive factors in estimating the odds of death. Moreover, RDW-SD is a strong independent predictor of infection severity and death in COVID-19 patients. An RDW-SD value > 47 indicated severe disease and a high risk of death.

It should be emphasized that during the first (univariate) step of our analysis, insulin and metformin administration were revealed to be insignificant in terms of mortality odds modulation. While this does not necessarily mean that insulin and metformin play no role in such predictions (as will be shown later in the discussion), it could be rightfully pointed out that insulin and metformin could not be used, on their own, in the process of estimating the risk of death in these patients.

In the next step of the analysis (deriving a multivariate model, Figure 3), the factors that were used in the previous step were all included in the initial pool of candidates for predictors of death. Subsequently, they were discarded one by one in a stepwise manner based on how much information they brought to the classifying (death/survival) model. As the pool of rejected factors began to increase, all of these variables were re-checked whether they should be again included in the model classifying mortality status. The multivariate model derived in the process enables estimation of the odds of death based on five factors: LMWH treatment, age, RDW-SD, sex and K. Calculating the odds of death of a patient admitted to the ward could be performed by multiplying the baseline odds (denoted by the intercept) based on the aforementioned characteristics. The value of the intercept and OR associated with sex show that survival would be the most frequent outcome (odds << 1) in a typical patient (described in the figure description) suffering from both COVID-19 and diabetes, regardless of sex (women: odds = 0.009, men: odds = 0.009 ∙ 2.798 ≈ 0.025). Had two patients of the same outcome been compared, each one-year gap between them would render the older patient 6.9% (odds = 1.069) more likely to die during the hospitalization. Each increase in potassium (by 1 mmol/L) and RDW-SD (by 15%) would, likewise promote death (by 98% and 87.7%, respectively). The association of these factors with death may be explained with cellular damage due to oxidative stress caused by inflammation. While, in this state, K would be simply liberated from the cells, the RDW-SD increase would be associated with the increase in anisocytosis in the state of constant/transient anemia—not only caused by their lysis per se but also correlated with kidney damage due to the lack of erythropoietin secretion. An over 10-fold increase in the odds of death upon LMWH treatment does not indicate that LMWH promotes death—it should rather hint that the decision of its administration was made in case of patients of higher disease severity emerging from possible increase of D-dimers, which account for the increased fibrinolysis. The mentioned D-dimers have been posed as mortality predictors in COVID-19 patients [36]. Moreover, increase plasmatic concentration of D-dimers among T2DM patients was shown to be associated with increased risk of cardiovascular disease events, regardless of conventional risk factors or the treatment-wise factors [36]. However, to our knowledge, D-dimers have not been proven (due to not having been studied) to pose as direct predictors of death in patients with T2DM nor patients with both T2DM and COVID-19. The last observation, already used while analyzing the baseline odds, is that men were of markedly higher odds of dying (in this model, OR = 2.798). While, as mentioned before, the odds in both sexes would be favoring survival in typical patients (64-year-old, 45.78% RDW-SD, 4.10 mmol/L K, no LMWH treatment), this sex-related difference would, epidemiologically, play an important role among the patients of higher age and disease severity. While this risk assessment model needs to undergo validation and comparison to different models in future studies to be taken more seriously, one is certain that the multivariate models, likewise to univariate, provide a hint that the administration of insulin and metformin is not a factor informative enough to be used in risk assessment of the entire COVID-19- and T2DM-positive population. Potential possibilities stemming from this information were revealed upon the last part of the analysis. The developed multivariate model allowed for the estimation of the chance of death based on LMWH treatment, age, RDW-SD, gender and K. Each increase in the patient’s age by one year increased the chance of death by 6.9%, each increase in potassium concentration by 1 mmol/L increased the chance of death by 98% and an increase in RDW-SD by 15% increased the chance of death by 87%, which was caused by cell damage by oxidative stress. LMWH was used in patients with advanced COVID-19, in whom an increase in D-dimer levels (increased fibrinolysis) was observed. The 10-fold increase in the risk of death after LMWH treatment was not due to the treatment itself, but to the stage of advancement of COVID-19. The association of gender with a higher risk of death concerned older patients with advanced disease. There was no gender effect observed for younger patients, aged 64.

The last part of the analysis explored the interactions between the variables (factors) in terms of changing the odds of death. What makes these interactions different to the convention used in the previous part of the study is that they explore whether any pair of factors (patient characteristics) has a multiplicative effect on the modulation of the odds of death by the third factor—a drug used during the hospitalization. LMWH and remdesivir were independent on each other in how they modulated the effect of metformin on the odds of death. Conversely, age and hsCRP interacted with each other, having a multiplicative effect on the difference in the odds of mortality between individuals who took insulin vs. the ones who did not. In the first observed interaction, remdesivir and LMWH treatments showed different patterns in affecting the odds of death, between patients administered with metformin and those under no such treatment. Upon analyzing the odds of death (N deaths/N survivals, Figure 4) it could be observed that the odds of death were different upon administration of remdesivir and LMWH, depending on whether the patient was under treatment with metformin. While metformin-administered patients showed an increasing pattern in the odds of death (LMWH and remdesivir > LMWH only > remdesivir only > neither LMWH nor remdesivir), such a pattern was not observed among the patients under no metformin treatment. One may argue about the novelty of this observation due to the fact that the treatment with remdesivir and LMWH blatantly shows the severity of the disease, thus implicating higher odds of death. However, to our knowledge, our study is the first one to show that this rationale was not universal for all of the COVID-19 T2DM patients. To gather more precise information on the odds of in-hospital death, before risk modeling, the entire population may need to be stratified in regards to metformin treatment and, perhaps, treatment with other antidiabetic agents as well. Another observation from this study was that metformin intake was associated with higher odds of death (0.20 vs. 0.10, Figure 4) compared to no intake among patients under no LMWH and remdesivir treatment. There is no possible way to discuss this matter referring to the literature since other studies did not report the odds of death in the same context as our study (three-way interaction). First and foremost, it is stated that metformin has both in vivo and in vitro effects on *SARS-CoV-2*, letting one assume there might be possibly different outcomes (and sets of its predictors) depending on metformin treatment [32]. Some studies on COVID-19 patients indicate lower likelihood of death upon metformin treatment [37,38,39]. However, DeFronzo et al. reported a lack of this association, but observed a markedly lower likelihood of heart failure among patients administered with metformin [38,40,41,42]. Analyzing interactions with inhibitors of dipeptydylpeptidase 4 (DPP-4i) may be a good choice for future analyses similar to ours, since this agent appears to have both direct and indirect effects on *SARS-CoV-2* infection. DPP-4i, being a gliptin [41] drug representative, owing to its anti-inflammatory action, could be hypothesized to indirectly (through lowering the CRP concentration) affect the severity of COVID-19 [38]. Another hypothesized indirect action of DPP-4i is combating the ‘cytokine storm’ through inhibiting the activation of TLR4 in the lung alveoli [5]. As *SARS-CoV-2* binds with these receptors, DPP-4i could help combat the pulmonary ‘cytokine storm’, leading to a decrease in lung injuries and collateral damage to other organs that could induce the state of multi-organ failure over the duration of COVID-19 [11,43]. Moreover, DPP-4i acts as a receptor for the *SARS-CoV-2* [13]; likewise, the drug binds with *MERS-CoV* [44]. This occurrence could have been associated with the observation of a lower concentration of soluble (in serum/plasma) DPP-4i among the diabetic COVID-19 patients [45]. So far, the idea behind using DPP-4i as a predictor of death/severity of the disease in the mentioned population could not be taken for granted due to the inconsistencies in the literature [46,47,48,49]. Likewise, remdesivir treatment had positive effects on the clinical improvement associated with the reduced risk of severe acute respiratory distress syndrome in need of intubation but it seemed not to affect mortality among COVID-19 patients [50]. In the presented publication, the analysis of interactions between variables (factors) in terms of the change in the probability of death showed that LMWH and remdesivir independently modulated the effect of metformin on the risk of death, while age and hsCRP interacted in their effect on the difference in the risk of death between people taking insulin and those not taking it. Metformin increased the risk of death the most in the group of people taking both LMWH and remdesivir. This observation was not demonstrated in the same group of people who did not take metformin. Additionally, age and hsCRP modulated the chance of death only in people who did not receive insulin.

However, DPP-4i could take part in interactions on which the information is scarce. Significant interaction of insulin with hsCRP and age hinted at different modulation of the odds of death by insulin, depending on these two other variables. This occurrence was due to fact that age and hsCRP did not significantly change the odds of death among patients administered with insulin (Table 3, Figure 5A,B), while the patients not administered with it showed a positive association between these odds and either hsCRP or age (Table 3, Figure 5A–C). This insulin-related difference between patients deepened the difference in odds between them when given more advanced age and/or higher hsCRP (Figure 5D,F). However, there is no universal answer to whether any of these groups would be more prone to showing a fatal outcome—it all is a matter of age and hsCRP (Figure 5F). Moreover, upon reaching a specific threshold of age and hsCRP, the odds would become of favor of death (e.g., more patients would die compared to the count of survivors) among the patients not administered with insulin. This interaction is not as complex as it could be, since age and hsCRP, although both simultaneously affecting the odds of death, had an isolated effect on it—hsCRP and age did not modulate the effect of each other on the odds (‘hsCRP*Age’ in Table 3) regardless of administration with insulin (‘Insulin*hsCRP*Age’ in Table 3). The cause of such a phenomenon, not discussed or mentioned in other studies, remains a mystery until validated and further analyzed on a bigger population, with possibly more factors brought into the model. A study [12] showed insulin treatment to be positively associated with the likelihood of death. However, the said study did not explore the possible effect of age on the insulin–mortality association. Perhaps a future model could employ both insulin–age and metformin–remdesivir–LMWH interactions. Hopefully, new research would investigate this matter on bigger retrospective data and/or a diabetic population not suffering from COVID-19 (assuming no COVID-19 outbreak in the future).

The third interaction was featured in this manuscript since it is associated with LMWH treatment, which is featured in both the multivariate model (Figure 3) and the aforementioned interaction (Figure 4). If one was to divide patients in the context of LMWH administration, the individuals under no such treatment would show constant odds of death equal to 0.202 regardless of age, meaning that the number of deceased patients would constitute about 1/5 of the survivors. Patients under LMWH treatment showed an increase in the odds with age, reaching the threshold which favors death at the age of approximately 85 years (odds > 1, thus N deaths > N survivors).

Before concluding the findings, study limitations need to be introduced. The first limitation comes from the rather low sample size (Figure 1). It should be emphasized that the data of all hospitalized T2DM patients from the Temporary COVID Hospital were employed for carrying out this study. Thus, we assumed the data to be randomly collected (in spite of the sample size), since all patients participated in the process. However, low sample size restricted us to study only up to two-way interactions and forming three-way interactions to be analyzed so as to avoid redundancy. Moreover, all of the observed interactions were not added to the multivariate model (Figure 3), having in mind that such a model would be highly prone to overfitting, thus would be biased with an increased false discovery rate. The sample size for such a model would need to be more than 1000 individuals (50–100 for every variable/interaction in the model), which exceeded the possibilities of our cooperation with this one hospital. The lack of comorbidities (seen in Table 1) in the initial set of variables was intentional so as to remove factors that could be so strongly associated with mortality that they would render other factors too weak to be spotted upon being analyzed in a population sample of such size. This choice was made upon assessing the comorbidity-associated frequencies and their statistics in regards to mortality (Table 1). Moreover, some patient features that could have had an impact on the observed were not registered upon creating this database in the times of COVID-19 onset. These features include BMI, diabetes duration, glycemic control, overall frailty, the stage of T2DM, and the severity of COVID-19. Since the decision on treatment with LMWH and remdesivir was made upon the admission of the study participants, there was no need for adjusting the models based on length of treatment with these agents. Our future study plans to gather the information from the patients regarding whether the treatment strategy for them changed after ending the hospitalization in the Temporary COVID-19 Hospital. Moreover, information on the post-hospitalization mortality in these individuals will be based on analyzing the national registry. Lastly, some may argue that the study shows neither goodness-of-fit metrics nor the classification quality of the model. While showing these properties of the model would be vital in a study that strived to determine the best death likelihood assessment model, our study focused on analyzing the models and interactions related to treatment. We explored the factors that may not even modulate the odds of risk per se, without their interactions with other patient characteristics (in this study: age, hsCRP and treatment with remdesivir or LMWH). Having these drawbacks in mind, we would like to encourage the readers to view this study as preliminary.

The insights from this study unfold to be rather peculiar, bringing some skepticism in the case of analyzing mortality risk with models based solely on logistic regression or (presumably) other regression methods. This study hints at possible caveats that could be encountered by simply using multivariate models without previously investigating whether the patterns of mortality changes associated with the predictors were affected by treatment. In this study, although insulin and/or metformin were not informative enough to be included in the multivariate assessment of the likelihood of death, the information about their administration revealed a contrast in how remdesivir and LMWH (in the case of metformin) compared to hsCRP and age (in the case of insulin) affected the odds of death in hospitalized T2DM patients suffering from COVID-19. Moreover, the association of LMWH treatment (one of the predictors in the multivariate model) with death was shown to be dependent on age. These observations not only show an importance of taking treatment into account when assessing death likelihood in the specific COVID-19 T2DM population, but hopefully may prove as grounds for future research into mortality modeling among T2DM patients. Although society has liberated itself from the grasp of the COVID-19 pandemic, the deleterious impact of the *SARS-CoV-2* infection may come with time in the form of newly studied post-COVID syndrome, leading to a sheer increase in the frequency of various comorbidities. If this time were to come, the analysis of interactions stemming from varying intakes of drugs may pose as a key to successful risk assessment, possibly saving thousands of lives and broadening our knowledge of other threats yet to come. In the further part of this research, we plan to analyze the mortality of patients included in the presented study in the second follow-up (after two years). We will also examine levels of early markers of kidney damage, neurological disorders and intravascular damage in patients who have had symptomatic COVID-19.

## 5. Conclusions

In a multivariate model, along with other multivariate-adjusted significant features (LMWH treatment, age, sex, K concentration), RDW-SD was associated with mortality among the patients suffering from COVID-19 and type 2 diabetes. For every 15% increase in RDW-SD, the odds of death increased by 87.7%.

Stratification by insulin administration revealed that age and hsCRP increased the odds of death exclusively among the patients who were not administered with insulin. Metformin intake was positively associated with death among those of low age and low hsCRP. Upon increase in both age and/or hsCRP above the threshold (mapped in Figure 5F), metformin intake started to be negatively associated with death. The impact of this effect kept rising with age and hsCRP.

Administration of remdesivir and/or LMWH changed the association between metformin and the odds of death from positive (if neither remdesivir nor LMWH were administered) to negative (if any of these drugs was administered). Moreover, remdesivir and LMWH had an additive effect on the magnitude of the pro-survival impact of metformin intake among the patients.

The association between LMWH administration and the odds of death changed from negative to positive with the increase in age.

The above findings ought to be taken with a pinch of salt until they have been validated with more sophisticated models (with these and other interactions), in a bigger population sample. Future research will, likewise, need to test these associations in a diabetic population not suffering from COVID-19.

Although metformin and insulin may not, per se, act as universal indicators of death in diabetic patients with COVID-19, their role could vary within the higher personalization of the risk assessment model (through adding and exploring their interactions with various patient characteristics). Such a practice, when utilized in large models, could provide a definite answer, cutting down the discussion of whether these agents are associated with death, when facing corroborating results from the literature. This conclusion applies regardless of whether the diabetic patients would be suffering from COVID-19 or not, since the studies into metformin and insulin in context of mortality lack the exploration of their interactions.

## Figures and Tables

**Table 1:** Baseline characteristics of COVID-19 patients at admission to the hospital and during the hospitalization.

**Demographic Variables (upon Hospital Admission)**					
**Variable**		**Category**	**Survivors (N = 333)**	**Non-Survivors (N = 107)**	***p***
Sex		Female	161 (48.35%)	37 (34.58%)	0.0127
Age [years]	Me (1Q–3Q)	-	70 (64–76)	75 (67–83)	0.0001
**Clinical Variables (upon Hospital Admission)**					
**Variable**		**Category**	**Survivors (N = 333)**	**Non-Survivors (N = 107)**	***p***
Metformin only		YES	87 (26.13%)	14 (13.08%)	0.0053
Insulin and other antidiabetic drugs		YES	32 (9.61%)	18 (16.82%)	0.0410
SpO_2_ ≤ 94%		YES	98 (51.04%)	31 (67.39%)	0.0456
Hemorrhage		YES	18 (5.41%)	16 (14.95%)	0.0013
Myocardial infarction		YES	47 (14.11%)	31 (28.97%)	<0.001
Heart failure		YES	72 (21.62%)	34 (31.78%)	0.0326
Prehospitalization oxygen therapy		YES	159 (47.75%)	69 (64.49%)	0.002
CKD		YES	60 (18.02%)	32 (29.91%)	0.0085
**Clinical Variables (after the Hospital Admission)**					
**Variable**		**Category**	**Survivors (N = 333)**	**Non-Survivors (N = 107)**	***p***
Revascularization: PCI or CABG		YES	6 (1.80%)	7 (6.54%)	0.0118
Features of pulmonary obstruction or pneumonia		YES	186 (55.86%)	85 (79.44%)	<0.001
Shock: hypovolemic, cardiogenic, septic		YES	13 (3.90%)	45 (42.06%)	<0.001
Hemorrhage: gastrointestinal, respiratory, intracranial, genital, urinary		YES	18 (5.41%)	16 (14.95%)	0.0013
Myocardial infarction		YES	6 (1.80%)	6 (5.61%)	0.0355
Acetylsalicylic acid		YES	89 (26.73%)	41 (38.32%)	0.022
**Laboratory Variables (upon Hospital Admission)**					
**Variable**			**Survivors**	**Non-Survivors**	***p***
		n	324	106	
RDW-SD [fL]		Me (1Q–3Q)	45.0 (41.60–49.10)	47.85 (43.40–52.40)	<0.001
		n	224	97	
LYMPH [count/µL]		Me (1Q–3Q)	1.11 (0.755–1.565)	0.76 (0.54–1.24)	<0.001
		n	105	29	
IL-6 [pl/mL]		Me (1Q–3Q)	15.3 (7.16–32.20)	56.3 (22.40–144.00)	<0.001
		n	242	103	
PCT [pg/mL]		Me (1Q–3Q)	0.10 (0.04–0.25)	0.31 (0.15–1.10)	<0.001
		n	101	76	
Albumin [mg/dL]		Me (1Q–3Q)	3.20 (2.90–3.60)	2.90 (2.50–3.20)	<0.001
		n	160	59	
Ferritin [µg/L]		Me (1Q–3Q)	507.40 (238.45–834.30)	850.00 (423.10–1368.10)	<0.001
		n	324	105	
CRP [mg/dL]		Me (1Q–3Q)	52.76 (12.85–112.45)	93.07 (0.62–487.40)	<0.001
		n	322	106	
K [mmol/L]		Me (1Q–3Q)	4.17 (3.70–4.62)	4.50 (4.10–4.80)	<0.001
		n	297	100	
Glucose [mg/dL]		Me (1Q–3Q)	152 (112.00–225.00)	196.50 (131.00–189.00)	0.0035
		n	323	106	
eGFR [mL/min/1.73 m^2^]		Me (1Q–3Q)	62.00 (41.00–81.00)	47.00 (26.00–69.00)	<0.001
		n	303	105	
Urea [mg/dL]		Me (1Q–3Q)	48.00 (34.00–78.00)	76.00 (52.00–113.00)	<0.001
		n	323	106	
Creatinine [mg/dL]		Me (1Q–3Q)	1.10 (0.84–1.52)	1.47 (0.98–2.32)	<0.001
		n	201	80	
LDH [U/L]		Me (1Q–3Q)	322.00 (244.00–438.00)	494.00 (328.50–665.50)	<0.001
		n	206	79	
Troponin [ng/L]		Me (1Q–3Q)	18.65 (7.30–67.90)	61.90 (21.30–275.50)	<0.001

Abbreviations: CABG, coronary artery bypass grafting; CKD, chronic kidney disease; CRP, C-reactive protein concentration; eGFR, estimated glomerular filtration rate; IL-6, interleukin 6; LDH, lactate dehydrogenase activity; LYMPH, lymphocyte count; Me, median value; PCI, percutaneous coronary intervention; PCT, procalcitonin concentration; Q, quartile.

**Table 2:** The derived multi-effect model for estimating the odds of death in type 2 diabetic, COVID-19-positive patients.

Iteration: Stepwise Elimination (*p* Cut-Off for Inclusion/Exclusion: 0.05); Baseline Group: Female. No LMWH. K: 4.10 mmol/L. RDW-SD: 45.78. Aged 64 Years											
Effect/Interaction	Analyzed Cat.	Reference Cat.	β	β SE	Wald Stat.	β −95% CI	β 95% CI	Est. Effect	Est. Effect −95% CI	Est. Effect 95% CI	*p*
Intercept (baseline odds)	-	-	−4.768	0.738	41.783	−6.213	−3.322	0.009	0.002	0.036	<0.001
K. centered at 4.10 mmol/L (OR)	-	-	0.683	0.254	7.257	0.186	1.180	1.980	1.205	3.255	0.007
Sex (OR)	Male	Female	1.029	0.417	6.090	0.212	1.846	2.798	1.236	6.337	0.014
log_1.15_(RDW-SD). centered at 27.36 (OR)	-	-	0.629	0.207	9.216	0.223	1.036	1.877	1.250	2.817	0.002
Age. centered at 64 years old (OR)	-	-	0.067	0.021	10.065	0.025	0.108	1.069	1.026	1.114	0.002
LMWH (OR)	1	0	2.325	0.602	14.906	1.145	3.505	10.223	3.141	33.272	<0.001

Abbreviations: Cat., category; CI, confidence interval; Est., estimated; LMWH, low-molecular-weight heparin; OR, odds ratio.

**Table 3:** Description of effects and their significant interactions in building a model to determine the modification of the impact of metformin intake on the likelihood of death in patients taking LMWH and/or remdesivir. (**A**) Reference group: no insulin administration, aged 64, with hsCRP 48.88. (**B**) Reference group: insulin administration, aged 64, with hsCRP 48.88.

**A. Reference Group: No Insulin Administration, Aged 64, with hsCRP 48.88**										
**Effect/Interaction**	**Description of the Analyzed Effect/Interaction**	**β**	**β SE**	**Wald Stat.**	**β −95% CI**	**β 95% CI**	**Est. Effect/Interaction**	**Est. Effect/Interaction −95% CI**	**Est. Effect/Interaction 95% CI**	***p***
Intercept	Odds of death for a patient not administered with insulin, aged 64, with hsCRP 48.88 mg/L	−2.026	0.224	81.551	−2.465	−1.586	**0.1319**	0.0850	0.2047	**<0.001**
Insulin	Modulation of odds by insulin administration at age 64 and hsCRP 48.88 mg/L	1.152	0.355	10.554	0.457	1.847	**3.1647**	1.5793	6.3414	**0.001**
hsCRP (centered at 48.88)	Change in odds upon each subsequent 1-unit increase in hsCRP	0.006	0.001	17.667	0.003	0.009	**1.0061**	1.0033	1.0090	**<0.001**
Age (centered at 64)	Change in odds upon each subsequent 1-year increase in age	0.059	0.014	17.278	0.031	0.087	**1.0608**	1.0317	1.0907	**<0.001**
Insulin*hsCRP	Fold difference in how administration with insulin would change the odds of death, upon each subsequent 1-unit increase in hsCRP	−0.007	0.003	5.720	−0.012	−0.001	**0.9932**	0.9877	0.9988	**0.017**
Insulin*Age	Fold difference in how administration with insulin would change the odds of death, upon each subsequent 1-year increase in age	−0.056	0.023	5.706	−0.102	−0.010	**0.9457**	0.9034	0.9900	**0.017**
ǂ hsCRP*Age	Fold change in how age and hsCRP modulated each other in changing the odds of death	−0.00009	0.00015	0.389	−0.00038	0.00020	0.9999	0.9996	1.0002	0.533
ǂ Insulin*hsCRP*Age	Fold change in how hsCRP and hsCRP modulated the impact of each other on changing the impact of insulin on the odds of death	0.00040	0.00029	1.978	−0.00016	0.00096	1.0004	0.9998	1.0010	0.160
**B. Reference Group: Insulin Administration, Aged 64, with hsCRP 48.88**										
**Effect/Interaction**	**Description of the Analyzed Effect/Interaction**	**β**	**β SE**	**Wald Stat.**	**β −95% CI**	**β 95% CI**	**Est. Effect/Interaction**	**Est. Effect/Interaction −95% CI**	**Est. Effect/Interaction 95% CI**	***p***
Intercept	Odds of death for a patient administered with insulin, aged 64, with hsCRP 48.88 mg/L	−0.874	0.275	10.119	−1.412	−0.335	**0.4174**	0.2437	0.7151	**0.001**
Insulin	Modulation of odds by the lack of insulin administration, at age 64 and hsCRP 48.88 mg/L	−1.152	0.355	10.554	−1.847	−0.457	**0.3160**	0.1577	0.6332	**0.001**
hsCRP (centered at 48.88)	Change in odds upon each subsequent 1-unit increase in hsCRP	−0.001	0.002	0.079	−0.005	0.004	0.9993	0.9946	1.0041	0.779
Age (centered at 64)	Change in odds upon each subsequent 1-year increase in age	0.003	0.019	0.031	−0.033	0.040	1.0033	0.9675	1.0404	0.861
Insulin*hsCRP	Fold difference in how the lack of administration with insulin would change the odds of death, upon each subsequent 1-unit increase in hsCRP	0.007	0.003	5.720	0.001	0.012	**1.0068**	1.0012	1.0124	**0.017**
Insulin*Age	Fold difference in how the lack of administration with insulin would change the odds of death, upon each subsequent 1-year increase in age	0.056	0.023	5.706	0.010	0.102	**1.0574**	1.0101	1.1069	**0.017**
ǂ hsCRP*Age	Fold change in how age and hsCRP modulated each other in changing the odds of death	0.00031	0.00024	1.605	−0.00017	0.00079	1.0003	0.9998	1.0008	0.205
ǂ Insulin*hsCRP*Age	Fold change in how hsCRP and hsCRP modulated the impact of each other on changing the impact of insulin on the odds of death	−0.00040	0.00029	1.978	−0.00096	0.00016	0.9996	0.9990	1.0002	0.160

‘X*Y’ terms denote interactions between variables (effects). ǂ denotes interactions that were explored upon the addition of both of them to the model. *p*-values lower than 0.05 and their corresponding ORs are marked **in bold**. Abbreviations: CI, confidence interval; hsCRP, high-sensitivity C-reactive protein; SE, standard error.

**Table 4:** Insights from a model aimed to assess the modulation of the association of LMWH treatment with the odds of death among COVID-19-positive diabetic patients by age.

**A. Reference Group: No LMWH Administration, Aged 64**										
**Effect/Interaction**	**Description of the Analyzed Effect/Interaction**	**β**	**β SE**	**Wald Stat.**	**β −95% CI**	**β 95% CI**	**Est. Effect/Interaction**	**Est. Effect/Interaction −95% CI**	**Est. Effect/Interaction 95% CI**	***p***
Intercept	Odds of death for a patient not administered with LMWH, aged 64	−2.036	0.293	48.338	−2.610	−1.462	0.131	0.074	0.232	**<0.001**
LMWH	Modulation of odds by LMWH administration, at age 64	0.785	0.343	5.235	0.113	1.457	2.192	1.119	4.295	**0.022**
Age (centered at 64)	Change in odds upon each subsequent 1-year increase in age	−0.006	0.022	0.073	−0.048	0.037	0.994	0.953	1.037	0.788
LMWH*Age	Fold difference in how administration with LMWH would change the odds of death, upon each subsequent 1-year increase in age	0.059	0.025	5.472	0.010	0.109	1.061	1.010	1.115	**0.019**
**B. Reference Group: LMWH Administration, Aged 64**										
**Effect/Interaction**	**Description of the Analyzed Effect/Interaction**	**β**	**β SE**	**Wald Stat.**	**β −95% CI**	**β 95% CI**	**Est. Effect/Interaction**	**Est. Effect/Interaction −95% CI**	**Est. Effect/Interaction 95% CI**	***p***
Intercept	Odds of death for a patient administered with LMWH, aged 64	−1.251	0.179	49.031	−1.602	−0.901	0.286	0.202	0.406	**<0.001**
LMWH	Modulation of odds by the lack of LMWH administration, at age 64	−0.785	0.343	5.235	−1.457	−0.113	0.456	0.233	0.894	**0.022**
Age (centered at 64)	Change in odds upon each subsequent 1-year increase in age	0.053	0.013	16.461	0.028	0.079	1.055	1.028	1.083	**<0.001**
LMWH*Age	Fold difference in how the lack of administration with LMWH would change the odds of death, upon each subsequent 1-year increase in age	−0.059	0.025	5.472	−0.109	−0.010	0.942	0.897	0.990	**0.019**

‘X*Y’ terms denote interactions between variables (effects). Values of the ‘Age’ variable were centered at 60 years of age. *p*-values lower than 0.05 are marked **in bold**. Abbreviations: CI, confidence interval; SE, standard error; LMWH, low-molecular-weight heparin.

## Data Availability

Access to the data is possible after sending an inquiry to the corresponding author by e-mail.

## Appendix A

**Table A1:** Results from the univariate analysis of the associations of the selected effects (variables) on the odds of death among COVID-19 patients suffering from type 2 diabetes.

Effect	Analyzed Cat.	Reference Cat.	OR	OR −95% CI	OR 95% CI	*p*
SpO_2_ ≤ 94 on room air	Yes	No	1.982	1.006	3.906	0.048
Sex	Male	Female	1.771	1.126	2.785	0.013
Insulin	Yes	No	1.364	0.832	2.236	0.219
Metformin	Yes	No	0.716	0.459	1.115	0.139
LMWH	Yes	No	3.506	1.972	6.234	<0.001
Acetylsalicylic acid	Yes	No	1.703	1.076	2.696	0.023
Convalescent plasma	Yes	No	1.618	0.878	2.981	0.123
Remdesivir	Yes	No	1.209	0.659	2.216	0.540
Age	-	-	1.038	1.017	1.060	<0.001
hsCRP	-	-	1.004	1.002	1.006	0.001
Na	-	-	1.026	0.988	1.066	0.184
K	-	-	1.886	1.399	2.543	<0.001
log_1.15_(RDW-SD)	-	-	1.538	1.235	1.915	<0.001
log_2_(Creatinine)	-	-	1.537	1.197	1.974	0.001
log_2_(Urea)	-	-	1.877	1.444	2.439	<0.001

Abbreviations: CI, confidence interval; OR, odds ratio. The visualization of this table (without the *p*-values) is given in Figure 2.

**Table A2:** The process of derivation of the multivariate model through stepwise elimination (cut-off *p* = 0.05).

Step	Effect	Wald Statistic	Wald Test *p*	Score Statistic	Score Test *p*	Effect Status
1	SpO_2_ ≤ 94 on room air	0.280	0.597			In the model
	Sex	5.731	0.017			In the model
	Insulin	0.550	0.458			In the model
	Metformin	<0.001	0.986			Excluded in this step
	LMWH	15.447	<0.001			In the model
	Acetylsalicylic acid	0.609	0.435			In the model
	Convalescent plasma	0.153	0.695			In the model
	Remdesivir	0.188	0.665			In the model
	Age	9.492	0.002			In the model
	hsCRP	1.822	0.177			In the model
	K	4.818	0.028			In the model
	log_2_(RDW-SD)	8.273	0.004			In the model
	log_2_(Creatinine)	0.426	0.514			In the model
2	SpO_2_ ≤ 94 on room air	0.282	0.596			In the model
	Sex	5.816	0.016			In the model
	Insulin	0.556	0.456			In the model
	log_2_(Creatinine)	0.433	0.511			In the model
	LMWH	15.510	<0.001			In the model
	Acetylsalicylic acid	0.610	0.435			In the model
	Convalescent plasma	0.154	0.695			Excluded in this step
	Remdesivir	0.188	0.664			In the model
	Age	9.684	0.002			In the model
	hsCRP	1.859	0.173			In the model
	K	4.817	0.028			In the model
	log_2_(RDW-SD)	8.299	0.004			In the model
	Metformin			0.000	0.986	Excluded in the previous step(s)
3	SpO_2_ ≤ 94 on room air	0.242	0.623			Excluded in this step
	Sex	5.731	0.017			In the model
	Insulin	0.483	0.487			In the model
	log_2_(Creatinine)	0.375	0.540			In the model
	LMWH	15.773	<0.001			In the model
	Acetylsalicylic acid	0.545	0.460			In the model
	log_2_(RDW-SD)	8.199	0.004			In the model
	Remdesivir	0.248	0.618			In the model
	Age	9.718	0.002			In the model
	hsCRP	1.924	0.165			In the model
	K	5.701	0.017			In the model
	Convalescent plasma			0.154	0.695	Excluded in the previous step(s)
	Metformin			0.001	0.981	Excluded in the previous step(s)
4	K	5.537	0.019			In the model
	Sex	5.628	0.018			In the model
	Insulin	0.602	0.438			In the model
	log_2_(Creatinine)	0.314	0.575			In the model
	LMWH	15.516	<0.001			In the model
	Acetylsalicylic acid	0.464	0.496			In the model
	log_2_(RDW-SD)	8.024	0.005			In the model
	Remdesivir	0.230	0.631			Excluded in this step
	Age	9.803	0.002			In the model
	hsCRP	1.686	0.194			In the model
	SpO_2_ ≤ 94 on room air			0.242	0.622	Excluded in the previous step(s)
	Convalescent plasma			0.113	0.736	Excluded in the previous step(s)
	Metformin			0.002	0.961	Excluded in the previous step(s)
5	K	5.740	0.017			In the model
	Sex	5.535	0.019			In the model
	Insulin	0.668	0.414			In the model
	log_2_(Creatinine)	0.299	0.584			Excluded in this step
	LMWH	15.916	<0.001			In the model
	Acetylsalicylic acid	0.566	0.452			In the model
	log_2_(RDW-SD)	7.903	0.005			In the model
	hsCRP	1.750	0.186			In the model
	Age	9.697	0.002			In the model
	Remdesivir			0.231	0.631	Excluded in the previous step(s)
	SpO_2_ ≤ 94 on room air			0.225	0.635	Excluded in the previous step(s)
	Convalescent plasma			0.167	0.683	Excluded in the previous step(s)
	Metformin			0.003	0.957	Excluded in the previous step(s)
6	K	7.608	0.006			In the model
	Sex	6.115	0.013			In the model
	Insulin	0.745	0.388			In the model
	Age	10.004	0.002			In the model
	LMWH	15.954	<0.001			In the model
	Acetylsalicylic acid	0.513	0.474			Excluded in this step
	log_2_(RDW-SD)	8.569	0.003			In the model
	hsCRP	1.815	0.178			In the model
	log_2_(Creatinine)			0.300	0.584	Excluded in the previous step(s)
	Remdesivir			0.215	0.643	Excluded in the previous step(s)
	SpO_2_ ≤ 94 on room air			0.170	0.680	Excluded in the previous step(s)
	Convalescent plasma			0.114	0.736	Excluded in the previous step(s)
	Metformin			0.001	0.972	Excluded in the previous step(s)
7	K	7.387	0.007			In the model
	Sex	5.872	0.015			In the model
	Insulin	0.459	0.498			Excluded in this step
	Age	9.772	0.002			In the model
	LMWH	15.713	<0.001			In the model
	hsCRP	1.937	0.164			In the model
	log_2_(RDW-SD)	8.993	0.003			In the model
	Acetylsalicylic acid			0.515	0.473	Excluded in the previous step(s)
	log_2_(Creatinine)			0.244	0.621	Excluded in the previous step(s)
	Remdesivir			0.315	0.575	Excluded in the previous step(s)
	SpO_2_ ≤ 94 on room air			0.104	0.747	Excluded in the previous step(s)
	Convalescent plasma			0.080	0.777	Excluded in the previous step(s)
	Metformin			0.007	0.934	Excluded in the previous step(s)
8	K	8.559	0.003			In the model
	Sex	5.788	0.016			In the model
	log_2_(RDW-SD)	8.793	0.003			In the model
	Age	9.999	0.002			In the model
	LMWH	15.825	<0.001			In the model
	hsCRP	2.036	0.154			Excluded in this step
	Insulin			0.461	0.497	Excluded in the previous step(s)
	Acetylsalicylic acid			0.235	0.628	Excluded in the previous step(s)
	log_2_(Creatinine)			0.317	0.573	Excluded in the previous step(s)
	Remdesivir			0.347	0.556	Excluded in the previous step(s)
	SpO_2_ ≤ 94 on room air			0.184	0.668	Excluded in the previous step(s)
	Convalescent plasma			0.031	0.859	Excluded in the previous step(s)
	Metformin			0.024	0.878	Excluded in the previous step(s)
9	K	7.739	0.005			In the model
	Sex	6.134	0.013			In the model
	log_2_(RDW-SD)	9.450	0.002			In the model
	Age	9.779	0.002			In the model
	LMWH	16.552	<0.001			In the model
	hsCRP			2.070	0.150	Excluded in the previous step(s)
	Insulin			0.556	0.456	Excluded in the previous step(s)
	Acetylsalicylic acid			0.288	0.592	Excluded in the previous step(s)
	log_2_(Creatinine)			0.385	0.535	Excluded in the previous step(s)
	Remdesivir			0.442	0.506	Excluded in the previous step(s)
	SpO_2_ ≤ 94 on room air			0.001	0.970	Excluded in the previous step(s)
	Convalescent plasma			0.095	0.758	Excluded in the previous step(s)
	Metformin			0.171	0.679	Excluded in the previous step(s)

The Wald test was utilized in assessing the value of the factors to be included in the model. The score test (Lagrange multiplier test) was used to check if, at any step, the previously excluded factors should be re-included into the current model. The final model is described in Table 2 and visualized in Figure 3.

**Table A3:** A list of all possible second-degree (two-way) interactions composed of the selected variables (effects, featured in Table A1 and Table A2, inter alia) in context of their significance (compared to the naïve model—LR type 1 test) in modulating the odds of death among COVID-19-positive individuals suffering from type 2 diabetes.

Effect 1	Effect 2	χ^2^	*p* (LR Test)
Metformin	Remdesivir	10.257	**0.0014**
LMWH	Metformin	5.6121	**0.0178**
hsCRP	Insulin	5.3447	**0.0208**
LMWH	Age	5.2361	**0.0221**
Age	Insulin	4.368	**0.0366**
K	Remdesivir	3.9539	**0.0468**
log_2_(Creatinine)	Insulin	3.8179	0.0507
LMWH	Insulin	3.743	0.053
Convalescent plasma	Metformin	3.7268	0.0535
K	SpO_2_ ≤ 94 on room air	3.7061	0.0542
hsCRP	SpO_2_ ≤ 94 on room air	3.037	0.0814
log2(RDW-SD)	Age	2.9078	0.0882
Metformin	Insulin	2.796	0.0945
Sex	Remdesivir	2.7621	0.0965
log_2_(Creatinine)	Metformin	2.7294	0.0985
LMWH	log_2_(Creatinine)	2.7034	0.1001
log_2_(Urea)	Remdesivir	2.6518	0.1034
K	hsCRP	2.3899	0.1221
LMWH	K	2.1219	0.1452
Acetylsalicylic acid	Metformin	2.0447	0.1527
hsCRP	Metformin	2.0331	0.1539
K	log_2_(Urea)	1.9158	0.1663
log_2_(RDW-SD)	hsCRP	1.9001	0.1681
K	Metformin	1.5692	0.2103
log_2_(RDW-SD)	SpO_2_ ≤ 94 on room air	1.5311	0.2159
LMWH	Remdesivir	1.5194	0.2177
Age	Metformin	1.4653	0.2261
Acetylsalicylic acid	Insulin	1.4478	0.2289
K	Acetylsalicylic acid	1.4253	0.2325
log_2_(Urea)	log_2_(RDW-SD)	1.3581	0.2439
log_2_(RDW-SD)	Metformin	1.1828	0.2768
LMWH	Acetylsalicylic acid	1.1417	0.2853
log_2_(Creatinine)	Remdesivir	1.1405	0.2856
log_2_(Urea)	Metformin	0.9777	0.3228
log_2_(RDW-SD)	Sex	0.9316	0.3344
Age	Convalescent plasma	0.9268	0.3357
LMWH	SpO_2_ ≤ 94 on room air	0.8762	0.3493
hsCRP	Convalescent plasma	0.8687	0.3513
hsCRP	Remdesivir	0.8583	0.3542
Acetylsalicylic acid	Remdesivir	0.8463	0.3576
Sex	Acetylsalicylic acid	0.8111	0.3678
LMWH	hsCRP	0.7506	0.3863
SpO_2_ ≤ 94 on room air	Remdesivir	0.7191	0.3964
log_2_(Urea)	Insulin	0.6931	0.4051
K	log_2_(Creatinine)	0.6634	0.4153
Convalescent plasma	Remdesivir	0.5695	0.4504
log_2_(Creatinine)	Convalescent plasma	0.5348	0.4646
Sex	Metformin	0.5297	0.4668
Age	hsCRP	0.5284	0.4673
log_2_(Creatinine)	Sex	0.5032	0.4781
log_2_(Creatinine)	Acetylsalicylic acid	0.4948	0.4818
Convalescent plasma	Insulin	0.4787	0.489
LMWH	Sex	0.3969	0.5287
K	log_2_(RDW-SD)	0.367	0.5446
K	Insulin	0.3392	0.5603
log_2_(Creatinine)	hsCRP	0.2903	0.59
Age	SpO_2_ ≤ 94 on room air	0.2817	0.5956
log_2_(RDW-SD)	Acetylsalicylic acid	0.258	0.6115
Insulin	Remdesivir	0.2404	0.6239
log_2_(Urea)	SpO_2_ ≤ 94 on room air	0.2237	0.6362
Age	Acetylsalicylic acid	0.2162	0.6419
LMWH	log_2_(Urea)	0.2161	0.642
SpO_2_ ≤ 94 on room air	Insulin	0.2128	0.6446
Age	Sex	0.2001	0.6547
Sex	SpO_2_ ≤ 94 on room air	0.1967	0.6574
Age	log_2_(Creatinine)	0.1935	0.66
LMWH	Convalescent plasma	0.1814	0.6702
log_2_(Creatinine)	SpO_2_ ≤ 94 on room air	0.1459	0.7025
hsCRP	Sex	0.1422	0.7061
log_2_(Urea)	log_2_(Creatinine)	0.1385	0.7098
log_2_(RDW-SD)	Insulin	0.1155	0.734
SpO_2_ ≤ 94 on room air	Convalescent plasma	0.1132	0.7365
SpO_2_ ≤ 94 on room air	Metformin	0.09	0.7642
log_2_(Urea)	hsCRP	0.0846	0.7711
K	Convalescent plasma	0.0605	0.8058
Acetylsalicylic acid	Convalescent plasma	0.0507	0.8218
Acetylsalicylic acid	SpO_2_ ≤ 94 on room air	0.0459	0.8303
Age	Remdesivir	0.0427	0.8363
log_2_(RDW-SD)	Convalescent plasma	0.0339	0.8538
hsCRP	Acetylsalicylic acid	0.0254	0.8733
K	Sex	0.0216	0.8831
log_2_(RDW-SD)	log_2_(Creatinine)	0.0209	0.8851
log_2_(Urea)	Acetylsalicylic acid	0.0186	0.8914
log_2_(RDW-SD)	Remdesivir	0.015	0.9026
K	Age	0.0086	0.9262
log_2_(Urea)	Sex	0.0066	0.935
Sex	Convalescent plasma	0.0041	0.9488
log_2_(Urea)	Age	0.0023	0.9621
Sex	Insulin	0.0019	0.9654
LMWH	log_2_(RDW-SD)	0.0016	0.9678
log_2_(Urea)	Convalescent plasma	0.0009	0.9767

Significant findings (*p* < 0.05) are marked **in bold**.

## References

[1] COVID-19 Cases|WHO COVID-19 Dashboard. https://data.who.int/dashboards/covid19/cases?n=c.

[2] Raman B., Bluemke D.A., Lüscher T.F., Neubauer S. (2022). Long COVID: Post-Acute Sequelae of COVID-19 with a Cardiovascular Focus. Eur. Heart J..

[3] Singh A.K., Khunti K. (2022). COVID-19 and Diabetes. Annu. Rev. Med..

[4] Steenblock C., Hassanein M., Khan E.G., Yaman M., Kamel M., Barbir M., Lorke D.E., Rock J.A., Everett D., Bejtullah S. (2022). Diabetes and COVID-19: Short- and Long-Term Consequences. Horm. Metab. Res..

[5] Nag S., Mandal S., Mukherjee O., Mukherjee S., Kundu R. (2023). DPP-4 Inhibitors as a Savior for COVID-19 Patients with Diabetes. Future Virol..

[6] Singh A.K., Gillies C.L., Singh R., Singh A., Chudasama Y., Coles B., Seidu S., Zaccardi F., Davies M.J., Khunti K. (2020). Prevalence of Co-morbidities and Their Association with Mortality in Patients with COVID-19: A Systematic Review and Meta-analysis. Diabetes Obes. Metab..

[7] Barron E., Bakhai C., Kar P., Weaver A., Bradley D., Ismail H., Knighton P., Holman N., Khunti K., Sattar N. (2020). Associations of Type 1 and Type 2 Diabetes with COVID-19-Related Mortality in England: A Whole-Population Study. Lancet Diabetes Endocrinol..

[8] Bode B., Garrett V., Messler J., McFarland R., Crowe J., Booth R., Klonoff D.C. (2020). Glycemic Characteristics and Clinical Outcomes of COVID-19 Patients Hospitalized in the United States. J. Diabetes Sci. Technol..

[9] Tittel S.R., Rosenbauer J., Kamrath C., Ziegler J., Reschke F., Hammersen J., Mönkemöller K., Pappa A., Kapellen T., Holl R.W. (2020). Did the COVID-19 Lockdown Affect the Incidence of Pediatric Type 1 Diabetes in Germany?. Diabetes Care.

[10] Holman N., Knighton P., Kar P., O’Keefe J., Curley M., Weaver A., Barron E., Bakhai C., Khunti K., Wareham N.J. (2020). Risk Factors for COVID-19-Related Mortality in People with Type 1 and Type 2 Diabetes in England: A Population-Based Cohort Study. Lancet Diabetes Endocrinol..

[11] Alshnbari A., Idris I. (2022). Can Sodium-Glucose Co-Transporter-2 (SGLT-2) Inhibitor Reduce the Risk of Adverse Complications Due to COVID-19?—Targeting Hyperinflammation. Curr. Med. Res. Opin..

[12] Varghese E., Samuel S.M., Liskova A., Kubatka P., Büsselberg D. (2021). Diabetes and Coronavirus (*SARS-CoV-2*): Molecular Mechanism of Metformin Intervention and the Scientific Basis of Drug Repurposing. PLoS Pathog..

[13] Li Y., Zhang Z., Yang L., Lian X., Xie Y., Li S., Xin S., Cao P., Lu J. (2020). The *MERS-CoV* Receptor DPP4 as a Candidate Binding Target of the *SARS-CoV-2* Spike. iScience.

[14] Zelniker T.A., Wiviott S.D., Raz I., Im K., Goodrich E.L., Bonaca M.P., Mosenzon O., Kato E.T., Cahn A., Furtado R.H.M. (2019). SGLT2 Inhibitors for Primary and Secondary Prevention of Cardiovascular and Renal Outcomes in Type 2 Diabetes: A Systematic Review and Meta-Analysis of Cardiovascular Outcome Trials. Lancet.

[15] Carino A., Moraca F., Fiorillo B., Marchianò S., Sepe V., Biagioli M., Finamore C., Bozza S., Francisci D., Distrutti E. (2020). Hijacking *SARS-CoV-2*/ACE2 Receptor Interaction by Natural and Semi-Synthetic Steroidal Agents Acting on Functional Pockets on the Receptor Binding Domain. Front. Chem..

[16] Baggen J., Jacquemyn M., Persoons L., Vanstreels E., Pye V.E., Wrobel A.G., Calvaresi V., Martin S.R., Roustan C., Cronin N.B. (2023). TMEM106B Is a Receptor Mediating ACE2-Independent *SARS-CoV-2* Cell Entry. Cell.

[17] Wang K., Chen W., Zhang Z., Deng Y., Lian J.-Q., Du P., Wei D., Zhang Y., Sun X.-X., Gong L. (2020). CD147-Spike Protein Is a Novel Route for *SARS-CoV-2* Infection to Host Cells. Signal Transduct. Target. Ther..

[18] Masre S.F., Jufri N.F., Ibrahim F.W., Abdul Raub S.H. (2021). Classical and Alternative Receptors for *SARS-CoV-2* Therapeutic Strategy. Rev. Med. Virol..

[19] Clinical Management of Severe Acute Respiratory Infection When Novel Coronavirus (nCoV) Infection Is Suspected: Interim Guidance, 12 January 2020. https://iris.who.int/handle/10665/332299.

[20] Levey A.S., Coresh J., Greene T., Stevens L.A., Zhang Y., Hendriksen S., Kusek J.W., Van Lente F. (2006). Using Standardized Serum Creatinine Values in the Modification of Diet in Renal Disease Study Equation for Estimating Glomerular Filtration Rate. Ann. Intern. Med..

[21] Alwani M., Yassin A., Al-Zoubi R.M., Aboumarzouk O.M., Nettleship J., Kelly D., AL-Qudimat A.R., Shabsigh R. (2021). Sex-based Differences in Severity and Mortality in COVID-19. Rev. Med. Virol..

[22] Muniyappa R., Gubbi S. (2020). COVID-19 Pandemic, Coronaviruses, and Diabetes Mellitus. Am. J. Physiol. Endocrinol. Metab..

[23] Rajpal A., Rahimi L., Ismail-Beigi F. (2020). Factors Leading to High Morbidity and Mortality of COVID-19 in Patients with Type 2 Diabetes. J. Diabetes.

[24] Norouzi M., Norouzi S., Ruggiero A., Khan M.S., Myers S., Kavanagh K., Vemuri R. (2021). Type-2 Diabetes as a Risk Factor for Severe COVID-19 Infection. Microorganisms.

[25] Solerte S.B., D’Addio F., Trevisan R., Lovati E., Rossi A., Pastore I., Dell’Acqua M., Ippolito E., Scaranna C., Bellante R. (2020). Sitagliptin Treatment at the Time of Hospitalization Was Associated With Reduced Mortality in Patients With Type 2 Diabetes and COVID-19: A Multicenter, Case-Control, Retrospective, Observational Study. Diabetes Care.

[26] Pitt B., Agarwal R., Anker S.D., Ruilope L.M., Rossing P., Ahlers C., Brinker M., Joseph A., Lambelet M., Lawatscheck R. (2022). Association of Finerenone Use With Reduction in Treatment-Emergent Pneumonia and COVID-19 Adverse Events Among Patients With Type 2 Diabetes and Chronic Kidney Disease. JAMA Netw. Open.

[27] Jung H.S., Choi J.W. (2023). Association between COVID-19 and Incidence of Cardiovascular Disease and All-Cause Mortality among Patients with Diabetes. Front. Endocrinol..

[28] Gazzaz Z.J. (2021). Diabetes and COVID-19. Open Life Sci..

[29] Li J., Huang D.Q., Zou B., Yang H., Hui W.Z., Rui F., Yee N.T.S., Liu C., Nerurkar S.N., Kai J.C.Y. (2021). Epidemiology of COVID-19: A Systematic Review and Meta-analysis of Clinical Characteristics, Risk Factors, and Outcomes. J. Med. Virol..

[30] Liu S., Zhang L., Weng H., Yang F., Jin H., Fan F., Zheng X., Yang H., Li H., Zhang Y. (2021). Association Between Average Plasma Potassium Levels and 30-Day Mortality During Hospitalization in Patients with COVID-19 in Wuhan, China. Int. J. Med. Sci..

[31] Mahroum N., Alghory A., Kiyak Z., Alwani A., Seida R., Alrais M., Shoenfeld Y. (2022). Ferritin—From Iron, through Inflammation and Autoimmunity, to COVID-19. J. Autoimmun..

[32] MCCarthy M.W. (2023). Metformin as a Potential Treatment for COVID-19. Expert. Opin. Pharmacother..

[33] Gama S. (2022). RDW Shows Prognostic Potential in Hospitalized Patients with COVID-19. J. Med. Virol..

[34] Soni M., Gopalakrishnan R. (2021). Significance of RDW in Predicting Mortality in COVID-19—An Analysis of 622 Cases. Int. J. Lab. Hematol..

[35] Pouladzadeh M., Safdarian M., Choghakabodi P.M., Amini F., Sokooti A. (2021). Validation of Red Cell Distribution Width as a COVID-19 Severity Screening Tool. Future Sci. OA.

[36] Soni M., Gopalakrishnan R., Vaishya R., Prabu P. (2020). D-Dimer Level Is a Useful Predictor for Mortality in Patients with COVID-19: Analysis of 483 Cases. Diabetes Metab. Syndr. Clin. Res. Rev..

[37] Bramante C.T., Ingraham N.E., Murray T.A., Marmor S., Hovertsen S., Gronski J., McNeil C., Feng R., Guzman G., Abdelwahab N. (2021). Metformin and Risk of Mortality in Patients Hospitalised with COVID-19: A Retrospective Cohort Analysis. Lancet Healthy Longev..

[38] Kim M.K., Jeon J.-H., Kim S.-W., Moon J.S., Cho N.H., Han E., You J.H., Lee J.Y., Hyun M., Park J.S. (2020). The Clinical Characteristics and Outcomes of Patients with Moderate-to-Severe Coronavirus Disease 2019 Infection and Diabetes in Daegu, South Korea. Diabetes Metab. J..

[39] Chen Y., Yang D., Cheng B., Chen J., Peng A., Yang C., Liu C., Xiong M., Deng A., Zhang Y. (2020). Clinical Characteristics and Outcomes of Patients With Diabetes and COVID-19 in Association With Glucose-Lowering Medication. Diabetes Care.

[40] DeFronzo R., Fleming G.A., Chen K., Bicsak T.A. (2016). Metformin-Associated Lactic Acidosis: Current Perspectives on Causes and Risk. Metabolism.

[41] Bugliani M., Syed F., Paula F.M.M., Omar B.A., Suleiman M., Mossuto S., Grano F., Cardarelli F., Boggi U., Vistoli F. (2018). DPP-4 Is Expressed in Human Pancreatic Beta Cells and Its Direct Inhibition Improves Beta Cell Function and Survival in Type 2 Diabetes. Mol. Cell Endocrinol..

[42] Ali N. (2020). Elevated Level of C-reactive Protein May Be an Early Marker to Predict Risk for Severity of COVID-19. J. Med. Virol..

[43] Choudhury A., Mukherjee S. (2020). In Silico Studies on the Comparative Characterization of the Interactions of *SARS-CoV-2* Spike Glycoprotein with ACE-2 Receptor Homologs and Human TLRs. J. Med. Virol..

[44] Vankadari N., Wilce J.A. (2020). Emerging COVID-19 Coronavirus: Glycan Shield and Structure Prediction of Spike Glycoprotein and Its Interaction with Human CD26. Emerg. Microbes Infect..

[45] Schlicht K., Rohmann N., Geisler C., Hollstein T., Knappe C., Hartmann K., Schwarz J., Tran F., Schunk D., Junker R. (2020). Circulating Levels of Soluble Dipeptidylpeptidase-4 Are Reduced in Human Subjects Hospitalized for Severe COVID-19 Infections. Int. J. Obes..

[46] Rakhmat I.I., Kusmala Y.Y., Handayani D.R., Juliastuti H., Nawangsih E.N., Wibowo A., Lim M.A., Pranata R. (2021). Dipeptidyl Peptidase-4 (DPP-4) Inhibitor and Mortality in Coronavirus Disease 2019 (COVID-19)—A Systematic Review, Meta-Analysis, and Meta-Regression. Diabetes Metab. Syndr. Clin. Res. Rev..

[47] Bonora B.M., Avogaro A., Fadini G.P. (2021). Disentangling Conflicting Evidence on DPP-4 Inhibitors and Outcomes of COVID-19: Narrative Review and Meta-Analysis. J. Endocrinol. Investig..

[48] Wargny M., Potier L., Gourdy P., Pichelin M., Amadou C., Benhamou P.-Y., Bonnet J.-B., Bordier L., Bourron O., Chaumeil C. (2021). Predictors of Hospital Discharge and Mortality in Patients with Diabetes and COVID-19: Updated Results from the Nationwide CORONADO Study. Diabetologia.

[49] Strollo R., Maddaloni E., Dauriz M., Pedone C., Buzzetti R., Pozzilli P. (2021). Use of DPP4 Inhibitors in Italy Does Not Correlate with Diabetes Prevalence among COVID-19 Deaths. Diabetes Res. Clin. Pract..

[50] Attena E., Caturano A., Annunziata A., Maraolo A.E., De Rosa A., Fusco F.M., Halasz G., Dall’Ospedale V., Conte M., Parisi V. (2023). Remdesivir treatment and clinical outcome in non-severe hospitalized COVID-19 patients: A propensity score matching multicenter Italian hospital experience. Eur. J. Clin. Pharmacol..

